# Fractal Analysis of Laplacian Pyramidal Filters Applied to Segmentation of Soil Images

**DOI:** 10.1155/2014/212897

**Published:** 2014-07-10

**Authors:** J. de Castro, F. Ballesteros, A. Méndez, A. M. Tarquis

**Affiliations:** ^1^Matemática Aplicada a las Tecnologías de la Información, E.T.S. de Ingenieros de Telecomunicación, Technical University of Madrid, Avenida Complutense 30, 28040 Madrid, Spain; ^2^Matemática Aplicada a la Ingeniería Técnica de Telecomunicación, E.T.S. de Ingeniería y Sistemas de Telecomunicación, Technical University of Madrid, Carretera de Valencia km. 7, 28031 Madrid, Spain; ^3^Matemática Aplicada a la Ingeniería Agronómica and CEIGRAM, E.T.S. de Ingenieros Agrónomos, Technical University of Madrid, Avenida Complutense 3, 28040 Madrid, Spain

## Abstract

The laplacian pyramid is a well-known technique for image processing in which local operators of many scales, but identical shape, serve as the basis functions. The required properties to the pyramidal filter produce a family of filters, which is unipara metrical in the case of the classical problem, when the length of the filter is 5. We pay attention to gaussian and fractal behaviour of these basis functions (or filters), and we determine the gaussian and fractal ranges in the case of single parameter *a*. These fractal filters loose less energy in every step of the laplacian pyramid, and we apply this property to get threshold values for segmenting soil images, and then evaluate their porosity. Also, we evaluate our results by comparing them with the Otsu algorithm threshold values, and conclude that our algorithm produce reliable test results.

## 1. Introduction

Image analysis involves many different tasks, such as identifying objects into images (segmentation), assigning labels to individual pixels by taking into account relevant information (classification), or extracting some meaning from the image as a whole (interpretation). The segmentation of soil images appears into Soil Science as a tool for the measurement of properties as well as for detecting and recognizing objects in soil [1–3].

Different methods have been used to segment soil images such as a simple binary threshold method [4] or multiple threshold method [5] and thresholds for typical and critical regions. Wang et al. [6] did a wide review of different segmentation methods applied in Geoscience. Other methods that appear to be most promising for soil applications are clustering methods and entropy-based methods [7–9].

Soil is not a continuous medium because soil is susceptible to changes from many influences: wetting, drying, compaction, plant growth, and so forth. So, the continuous soil models lead to approximate results only, and anomalous phenomena cannot be easily handled. It is known that pores in porous material are highly complex [10]. Their study and analysis have been usually avoided because of their difficulty.

Soil is formed from many constituents, and to represent it as a two-phase material, solid and pore, is often an oversimplification. The behaviour of water, gas, and organisms can affect it. A classification of pore models could be [11] (i) nonspatial, (ii) schematic, (iii) random set, (iv) fractal, and (v) other stochastic models. The fractal group has more models proposed and publications about.

Models of soil physical structure have been developed since the 1950s. Childs and Collisgeorge [12] introduced the cut-and-rejoin models of soil capillaries, which were modified by Marshall [13]. While many models of soil structure have been developed since then, most relate the structure to physical processes, generally ignoring heterogeneity, or assume simple pore-size distribution models.

More sophisticated approaches are [14] using a one-dimensional Markov chain model for horizontal soil, [15] proposing a two-dimensional fuzzy random model of soil pore structure, and [16] describing a network model to predict physical properties from topological parameters and fractal-based approaches like [17].

Our goal is to calculate the porosity of soil images. The proposed procedure for segmentation of soil micromorphological images is based on Laplacian pyramid algorithm [18], from which we compute a threshold that will binarize the original image, resulting with an image composed of continuous regions of pores (shown in black) and soil (shown in white). From this binarized soil image we compute an estimation to its porosity.

Another objective of this study is also to compare the results with those provided by the commonly used Otsu's algorithm [19] that is widely accepted as a good method to get an appropriate threshold.

## 2. Materials and Methods

### 2.1. Laplacian Pyramid

A multiresolution model consists of generating different versions of a given image by decreasing the initial resolution, which also means decreasing the initial size. This is achieved by a downsampling operator which must be associated with an appropriate filtering to avoid aliasing phenomena (downsampling theorem). Multiresolution approaches have been investigated for different purposes such as image segmentation and image compression [18]. In terms of image analysis, low resolution representations are convenient for global detection and recognition of image features while minute details can only be seen on high resolution images.

One usual property of images is that neighboring pixels are highly correlated. This property is inefficient to represent the complete image directly in terms of its pixel values, because most of the encoded information would be redundant. Burt and Adelson designed a technique, named Laplacian pyramid, for removing image correlation which combines characteristics of predictive and transform methods [18]. This technique is noncausal, and computations are relatively simple and local. The predicted value for each new pixel is computed as a local weighted average, using a unimodal weighting function centered on the pixel itself.

This pyramidal representation is useful for two important classes of computer graphics problems. The first class is composed of those tasks that involve analysis of existing images, such as merging images or interpolating to fill in missing data smoothly, become much more intuitive when we can manipulate easily visible local image features at several spatial resolutions. And the second, when we are synthesizing images, the pyramid becomes a multiresolution sketch pad. We can fill in the local spatial information at increasingly fine detail by specifying successive levels of a pyramid.

The first time that pyramidal structures were applied to multiresolution decompositions was at [18]. Later, the relationship to wavelets was realized shortly thereafter, because both decompositions are based on the idea of successive refinement: the image is obtained as a sum of an initial coarse version plus detail signals. One interesting thing to note about the pyramidal approach is that perfect reconstruction is possible; therefore it is a lossless data algorithm.

Pyramidal methods for multiresolution image analysis have been used since the 1970s. Early work in multiresolution image description was primarily motivated by a desire to reduce the computational cost of methods for image description and image matching. Later, multiresolution processing was generalized to computing multiple copies of an image by repeatedly summing nonoverlapping blocks of pixels and resampling until the image is reduced to a small number of pixels. Such a structure became known as a multiresolution pyramid [20].

Interest in multiresolution techniques for signal processing and analysis is increasing steadily [21]. An important instance of such a technique is the so-called pyramidal decomposition scheme. Our work uses a general axiomatic pyramidal decomposition scheme for soil image analysis. This scheme comprises the following ingredients.

- The pyramid consists of a finite number of levels such that the information content decreases towards higher levels.
- Each step towards a higher level is constituted by an information-reducing analysis operator, whereas each step towards a lower level is modeled by an information-preserving synthesis operator. One basic assumption is necessary: synthesis followed by analysis yields the identity operator, meaning that no information is lost by these two consecutive steps.

### 2.2. Fractal Dimension

The techniques based on fractals show promising results in the field of image understanding and visualization of high complexity data.

The high complexity of some images demands new techniques for understanding and analyzing them. The similarity of fractals and real world objects has been observed and studied from the very beginning. The fractal geometry became a tool for computer graphics and data visualization in the simulation of the real world. In order to perform visual analysis and comparisons between natural and synthetic scenes several techniques have been developed. After a period of qualitative experiments, fractal geometry began to be used for objective and accurate purposes: modeling images, evaluating their characteristics, analyzing their textures, and so forth.

Nowadays, there are a lot of fields where fractals appear [22]. First of all, we present some of the elementary ideas necessary to understand applications of fractal geometry in geo-information processing [23].

Fractal geometry theory deals with the behaviors of sets of points *S*, in the *n*-dimensional space *R* ^*n*^. Images, particularly soil images, are sets of points in *R* ^2^.

Mandelbrot defined a fractal as a shape made of parts similar to the whole in some way [24]. That definition is qualitative but not ambiguous, as it looks at the first glance. The main behavior of a fractal is its self-similarity. A set is called self-similar if it can be expressed as a union of sets, each of which is a reduced copy of the full set. More generally a set is said to be self-affine if it can be decomposed into subsets that can be linearly mapped into the full set. If the linear mapping is a rotation, translation, or isotropic dilatation the set is self-similar. The self-similar objects are particular cases of self-affine ones.

A fractal object is self-similar or self-affine at any scale. If the similarity is not described by deterministic laws stochastic resemblance criteria can be found. Such an object is said to be statistical self-similar. The natural fractal objects are statistically self-similar. A statistically self-similar fractal is by definition isotropic. To have a more precise, quantitative description of the fractal behavior of a set, a measure and a dimension are introduced. The rigorous mathematical description is done by Hausdorff's measure and dimension [25].

Let *S* ⊂ *R* ^*n*^ and *r* > 0, and a *δ*-cover of *S* is a collection of sets {*U*_*i*_ : *i* ∈ *I*} with diameter which is smaller than *δ*, such that

(1) $$\begin{array}{c} S\subset \underset{i\in I}{⋃}U_{i}\subset R^{n}\;\text{with}\;\;0<\left|U_{i}\right|<\delta , \end{array}$$

where *I* is a finite or countable index set and |·| represents the diameter of the *n*-dimension set, defined as

(2) $$\begin{array}{c} \left|U\right|=\sup\left\{\left|x-y\right|:x,y\in U\right\}. \end{array}$$

Also, let *R*_*δ*_(*S*) be the collection of all *δ*-covers of *S*; we can define

(3) $$\begin{array}{c} H_{\delta }^{r}\left(S\right)=\underset{R\in R_{\delta }}{\inf}\left\{\underset{i\in I}{\sum }{\left|U_{i}\right|}^{r}:R=\underset{i\in I}{⋃}U_{i}\right\}. \end{array}$$

Now, if in (3) we let *δ* decrease to zero, we get the Hausdorff measure of the set *S*, *H* ^*r*^(*S*):

(4) $$\begin{array}{c} H^{r}\left(S\right)=\underset{\delta \to 0}{\lim}H_{\delta }^{r}\left(S\right). \end{array}$$

The Hausdorff measure generalizes the definition of length, area, volume, and so on. *H*_*δ*_^*r*^(*S*) gives the volume of a set *S* as measured with a ruler of *δ* units.

There is an interesting property of the Hausdorff measure: If the Hausdorff dimension of the set *S* is *s*, then

(5) $$\begin{array}{c} H^{p}\left(S\right)=\{\begin{array}{cc} \infty & \text{if}\;\;p<s\\ 0 & \text{if}\;\;p>s. \end{array} \end{array}$$

**Figure 1:**
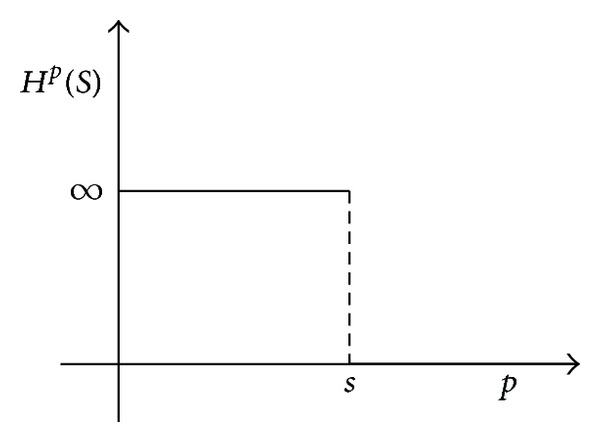
Hausdorff dimension of the set *S*.

So, the Hausdorff dimension of the set *S* ⊂ *R* ^*n*^ could be defined as

(6) $$\begin{array}{c} \dim_{H}S=\sup\left\{r:H^{r}\left(S\right)=\infty \right\}=\inf\left\{r:H^{r}\left(S\right)=0\right\} \end{array}$$

as we can see in Figure 1.

Then, the value of the parameter *r* for which the *r*-dimensional Hausdorff measure of the set jumps from zero to infinite is said to be the Hausdorff dimension, dim⁡_*H*_, of the set *S*.

A set is said to be fractal if its Hausdorff dimension strictly exceeds its topological dimension, dim⁡_*H*_ *S* > *n*.

Numerical evaluation of Hausdorff dimension is difficult because of the necessity to evaluate the infimum of the measure over all the coverings belongings to the set of interest. That is the reason to look for another definition for the dimension of a set. The box counting dimension allows the evaluation of the dimension of sets of points spread in an *n*-dimensional space and also gives possibilities for easy algorithmic implementation.

Given a set of points *S*, in a *n*-dimensional space *R* ^*n*^, and *N*_*δ*_ is the least number of sets of diameter at most *δ* that cover *S*, the box counting dimension, dim⁡_*B*_, is defined as

(7) $$\begin{array}{c} \dim_{B}S=\underset{\delta \to 0}{\lim}\frac{\log N_{\delta }}{\log1/\delta }. \end{array}$$

Depending on the geometry of the box and the modality to cover the set, several box counting dimensions can be defined using closed balls, cubes, and so on [25].

The equivalence of these definitions was proved. Also it was proved that these dimensions are inferior bounded by the Hausdorff dimension [26].

Fractal geometry provides a mathematical model for many complex objects found in nature, such as coastlines, mountains, and clouds [22, 24]. These objects are too complex to possess characteristic sizes and to be described by traditional Euclidean geometry. Fractal dimension has been applied in texture analysis and segmentation [27, 28]. There are different methods that have been proposed to estimate the fractal dimension. The three major categories are the box-counting methods, the variance methods, and the spectral methods. The box-counting dimension is the most frequently used for measurements in various application fields. The reason for its dominance lies in its simplicity and automatic computability.

### 2.3. Segmentation of Soil Images

Image segmentation is the process of partitioning an image into several regions, in order to be easier to analyze and work with.

In image segmentation the level to which the subdivision of an image into its constituent regions or objects is carried depending on the problem being solved. In other words, when the object of focus is separated, image segmentation should stop [29]. The main goal of segmentation is to divide an image into parts having strong correlation with areas of interest in the image.

We study the simplest problem, dividing the image into just only two parts, foreground and background, or object pixels and background pixels. The intensity values, continuity or discontinuity, color, texture, and other image characteristics are the origin of the different image segmentation techniques. Reference [9] is an exhaustive performance comparison of 40 selected methods put into groups according to histogram shape information, measurement space clustering, histogram entropy information, image attribute information, spatial information, and local characteristics.

So, some of the most important groups in image segmentation techniques are the threshold-based, the histogram-based, the edge-based, and the region based.

The threshold-based methods are based on pixels intensity values. The main goal here is to decide a threshold value *λ* to apply the rule:

(8) $$\begin{array}{c} g_{i+1}\left(x,y\right)=\{\begin{array}{cc} 0 & \text{if}\;\;g_{i}\left(x,y\right)<\lambda \\ 255 & \text{if}\;\;g_{i}\left(x,y\right)\geq \lambda , \end{array} \end{array}$$

where *g*_*i*+1_(*x*, *y*) is the new pixel value and *g*_*i*_(*x*, *y*) is the old pixel value. In other words, after choosing a threshold, then every pixel in the image is compared with this threshold, and if the pixel lies above the threshold it will be marked as foreground, and if it is below the threshold it will be marked as background. The histogram-based methods are also based on pixels' intensity values. Here, histogram bars help to find the clusters of pixels values. One of the most famous threshold-based methods is Otsu's method [19].

The edge-based methods show boundaries in the image, determining different regions where we have to decide if they are foreground or background. The boundaries are calculated analyzing high contrasts in intensity, color, or texture. On the other hand, an opposed point of view are the region-based methods divide the image into regions, searching for same textures, colors, or intensity values.

In soil science the porosity of a porous medium is defined by the ratio of the void area and the total bulk area. Therefore, porosity is a fraction whose numerical value is between 0 and 1, typically ranging from 0.005 to 0.015 for solid granite to 0.2 to 0.35 for sand. It may also be represented in percent terms by multiplying the number by 100. Porosity is a dimensionless quantity and can be reported either as a decimal fraction or as a percentage.

The total porosity of a porous medium is the ratio of the pore volume to the total volume of a representative sample of the medium. Assuming that the soil system is composed of three phases—solid, liquid (water), and gas (air)—where *V*_*s*_ is the volume of the solid phase, *V*_*l*_ is the volume of the liquid phase, *V*_*g*_ is the volume of the gaseous phase, *V*_*p*_ = *V*_*l*_ + *V*_*g*_ is the volume of the pores, and *V*_*t*_ = *V*_*s*_ + *V*_*l*_ + *V*_*g*_ is the total volume of the sample, then the total porosity of the soil sample, *p*_*t*_, is defined as follows:

(9) $$\begin{array}{c} p_{t}=\frac{V_{p}}{V_{t}}=\frac{V_{l}+V_{g}}{V_{s}+V_{l}+V_{g}}. \end{array}$$

Table 1 lists representative porosity ranges for various geologic materials [30]. In general, porosity values for unconsolidated materials lie in the range of 0.25–0.7 (i.e., 25%–70%). Coarse-textured soil materials (such as gravel and sand) tend to have a lower total porosity than fine-textured soils (such as silts and clays). Porosity values in soils are not a constant quantity because the soil, particularly clayey soil, alternately swells, shrinks, compacts, and cracks. The porosity of our test image, shown in Figure 10(a), is 0.284.

Our work applies image segmentation techniques to calculate the porosity of soil images. Also, we have compared our results with the Otsu image segmentation algorithm.

## 3. Methodology

### 3.1. Laplacian Pyramid

**Figure 2:**
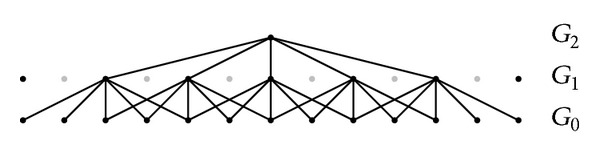
Representation of the one-dimensional Gaussian pyramid process.

**Figure 3:**
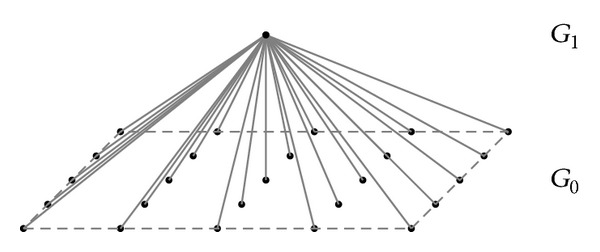
Representation of the two-dimensional Gaussian pyramid process.

The Laplacian pyramid representation expresses the original image as a sum of spatially band-passed images, while retaining local spatial information in each band. The Gaussian pyramid is created by low-pass-filtering an image *G*_0_ with a two-dimensional compact filter. The filtered image is then subsampled by removing every other pixel and every other row to obtain a reduced image *G*_1_. Graphical representations of these processes in one and two dimensions are given in Figures 2 and 3.

This process is repeated to form a Gaussian pyramid *G*_0_, *G*_1_, *G*_2_,…, *G*_*N*_:

(10) $$\begin{array}{c} G_{k}\left(i,j\right)=\underset{m}{\sum }\underset{n}{\sum }G_{k-1}\left(2i+m,2j+n\right)\;k=1,\ldots ,N. \end{array}$$

Expanding *G*_1_ to the same size as *G*_0_ and subtracting yields the band-passed image *L*_0_, a Laplacian pyramid *L*_0_, *L*_1_,…, *L*_*n*−1_ can be built containing band-passed images of decreasing size and spatial frequency:

(11) $$\begin{array}{c} L_{k}=G_{k}-G_{k+1}\;k=0,\ldots ,N-1, \end{array}$$

where the expanded image *G*_*k*_ is given by

(12) $$\begin{array}{c} G_{k}\left(i,j\right)=4\underset{m}{\sum }\underset{n}{\sum }w\left(m,n\right)G_{k-1}\left(\frac{2i+m}{2},\frac{2j+n}{2}\right). \end{array}$$

The original image can be reconstructed from the expanded bandpass images:

(13) G0=L0+G1=L0+L1+G2⋮=L0+L1+L2+⋯+LN−1+GN.

The Gaussian pyramid contains low-passed versions of the original *G*_0_ at progressively lower spatial frequencies, while the Laplacian pyramid consists of band-passed copies of the original image *G*_0_. Each Laplacian level contains the edges of a certain size and spans approximately an octave in spatial frequency.

### 3.2. Fractal Dimension: Box-Counting Dimension

Fractal dimension is a useful feature for texture segmentation, shape classification, and graphic analysis in many fields. The box-counting approach is one of the frequently used techniques to estimate the fractal dimension of an image.

There are several methods available to estimate the dimension of fractal sets. The Hausdorff dimension is the principal definition of fractal dimension. However, there are other definitions, like box-counting or box dimension, that is popular due to its relative ease of mathematical calculation and empirical estimation. The main idea to most definitions of fractal dimension is the idea of measurement at scale *δ*. For each *δ*, we measure a set ignoring irregularities of size less than *δ*, and then we see how these measurements behave as *δ* → 0. For example, if *F* is a plane curve (one of our filters), then *N*_*δ*_(*F*) might be the number of steps required by a pair of dividers set at length *δ* to traverse *F*. Then, the dimension of *F* is determined by the power law, if any exists, obeyed by *N*_*δ*_(*F*) as *δ* → 0. So, we might say that *F* has dimension *s* if a constant *s* exists so that

(14) $$\begin{array}{c} \delta^{s}\cdot N_{\delta }\left(F\right)≃1, \end{array}$$

where taking logarithms and limits when *δ* tends to 0; we get (7).

These formulae are appealing for computational or experimental purposes, since *s* can be estimated as the gradient of a log-log graph plotted over a suitable range of *δ*.

### 3.3. Kolmogorov-Smirnov Normality Test

In order to determine the normality interval we use the Kolmogorov-Smirnov normality test [31, 32], which is the most usual empirical distribution function test for normality.

For a data set *A* of *n* we make the contrast of a distribution function *F*_*n*_ from a theoretical distribution function *F*, using the statistic *D*_*n*_

(15) $$\begin{array}{c} D_{n}=D_{n}\left(F_{n},F\right)=\underset{x\in A}{\max}\left\{\left|F_{n}\left(x\right)-F\left(x\right)\right|\right\} \end{array}$$

that represents the distance between *F*_*n*_ and *F*.

For *n* large enough, the statistical distribution of *D*_*n*_ is close to the Kolmogorov-Smirnov distribution, *K*, which is tabulated for some significant values. Obviously, the assumption of normality is rejected with significance level 1 − *α*, if *D*_*n*_ > *D*_*n*,*α*_, with *P*(*K* ≤ *D*_*n*,*α*_) = *α*.

Let *D*_*n*,*α*_ be the K-S distribution percentiles. We reject at level 1 − *α* because if *n* is big enough, *D*_*n*_ = *K* and *α* = 0.01. Then, we reject small values of *D*_*n*_, so if |*F*_*n*_ − *F*| is smaller than the percentile *α*, we accept the hypothesis.

### 3.4. Otsu's Thresholding Method

The most common image segmentation methods are the histogram thresholding based, since thresholding is easy, fast, and economical in computation. For performing the image segmentation we need to calculate a threshold which will separate the objects and the background in our image. Since soil images are relatively simple when we just pay attention to void and bulk, so we are going to apply the global threshold technique, instead of more advanced variations (band thresholding, local thresholding, and multithresholding). The global thresholding technique consists of selecting one threshold value and applying it to the whole image.

The resultant image is a binary image where pixels that correspond to objects and background have values of 255 and 0, respectively. Quick and simple calculation is the main advantage of global thresholding.

Otsu's method searches for the threshold that minimizes the intraclass variance (or within class variance) *σ*_*W*_^2^(*t*) defined as the weighted sum of variances of the two classes:

(16) $$\begin{array}{c} \sigma_{w}^{2}\left(t\right)=\omega_{0}\left(t\right)\sigma_{0}^{2}\left(t\right)+\omega_{1}\left(t\right)\sigma_{1}^{2}\left(t\right), \end{array}$$

where *ω*_*i*_ are the probabilities of the two classes (foreground and background) separated by a threshold *t* and *σ*_*i*_^2^ are the variances of these both classes.

Otsu [19] proofed that minimizing the intraclass variance is the same as maximizing the interclass variance *σ*_*b*_^2^(*t*) defined as follows:

(17) $$\begin{array}{c} \sigma_{B}^{2}\left(t\right)=\sigma^{2}-\sigma_{W}^{2}\left(t\right)=\omega_{0}\left(t\right)\omega_{1}\left(t\right){\left[\mu_{0}\left(t\right)-\mu_{1}\left(t\right)\right]}^{2}, \end{array}$$

where *ω*_*i*_ are the class probabilities and *μ*_*i*_ the class means.

The class probabilities *ω*_0_(*t*) and *ω*_1_(*t*), and the class means *μ*_0_(*t*) and *μ*_1_(*t*), are computed as

(18) $$\begin{array}{c} \omega_{0}\left(t\right)=\overset{t}{\underset{0}{\sum }}p\left(i\right)\;\;\omega_{1}\left(t\right)=\underset{i>t}{\sum }p\left(i\right)\\ \mu_{0}\left(t\right)=\overset{t}{\underset{0}{\sum }}p\left(i\right)x\left(i\right)\;\;\mu_{1}\left(t\right)=\underset{i>t}{\sum }p\left(i\right)x\left(i\right), \end{array}$$

where *x*(*i*) is the centered value of the *i*th histogram bin.

The class probabilities and class means can be computed iteratively. This idea yields an effective algorithm.

Otsu's algorithm assumes just only two sets of pixel intensities, the foreground and the background, or void and bulk for soil images. The main idea of the Otsu's method is to minimize the weighted sum of within-class variances of the foreground and background pixels to establish an optimum threshold. It can be formulated as

(19) $$\begin{array}{c} \lambda_{\text{Otsu}}=\arg\;\min\left\{\omega_{0}\left(\lambda \right)\sigma_{0}^{2}\left(\lambda \right)+\omega_{1}\left(\lambda \right)\sigma_{1}^{2}\left(\lambda \right)\right\}, \end{array}$$

where the weights *ω*_*i*_(*λ*) are the probabilities of the two classes separated by the threshold *λ* and *σ*_*i*_^2^(*λ*) are the corresponding variances of these classes. Otsu's method gives satisfactory results when the values of pixels in each class are close to each other, as in soil images.

Let the pixels of a given picture be represented in 256 gray levels: 0,1, 2,…, 255. Let the number of pixels at level *i* be denoted by *n*_*i*_ and the total number of pixels by *N*. If we define *p*_*i*_ as *p*_*i*_ = *n*_*i*_/*N*, then *p*_*i*_ ≥ 0 and ∑*p*_*i*_ = 1. So, we have a probability distribution point of view.

The threshold at level *k* defines two classes: the foreground (*C*_0_) and the background (*C*_1_). Then, the probabilities of these classes and their means are

(20) $$\begin{array}{c} \omega_{0}=\text{Pr}\left(C_{0}\right)=\overset{k}{\underset{i=0}{\sum }}p_{i}=\omega \left(k\right),\\ \omega_{1}=\text{Pr}\left(C_{1}\right)=\overset{255}{\underset{i=k+1}{\sum }}p_{i}=1-\omega \left(k\right),\\ \mu_{0}=\overset{k}{\underset{i=0}{\sum }}i\text{Pr}\left(i\;|\;C_{0}\right)=\overset{k}{\underset{i=0}{\sum }}\frac{ip_{i}}{\omega_{0}}=\frac{\mu \left(k\right)}{\omega \left(k\right)},\\ \mu_{1}=\overset{255}{\underset{i=k+1}{\sum }}i\text{Pr}\left(i\;|\;C_{1}\right)=\overset{255}{\underset{i=k+1}{\sum }}\frac{ip_{i}}{\omega_{1}}=\frac{\mu_{T}-\mu \left(k\right)}{1-\omega \left(k\right)}, \end{array}$$

where

(21) $$\begin{array}{c} \omega \left(k\right)=\overset{k}{\underset{i=0}{\sum }}p_{i}\;\;\mu \left(k\right)=\overset{k}{\underset{i=0}{\sum }}ip_{i},\;\;\mu_{T}=\overset{255}{\underset{0}{\sum }}ip_{i}. \end{array}$$

We can easily verify that for any *k*: *ω*_0_ + *ω*_1_ = 1 and *ω*_0_ *μ*_0_ + *ω*_1_ *μ*_1_ = *μ*_*T*_. Now, we define the class variances as

(22) $$\begin{array}{c} \sigma_{0}^{2}=\overset{k}{\underset{i=0}{\sum }}{\left(i-\mu_{0}\right)}^{2}\text{Pr}\left(i\;|\;C_{0}\right)=\overset{k}{\underset{i=0}{\sum }}\frac{{\left(i-\mu_{0}\right)}^{2}p_{i}}{\omega_{0}},\\ \sigma_{1}^{2}=\overset{255}{\underset{i=k+1}{\sum }}{\left(i-\mu_{1}\right)}^{2}\text{Pr}\left(i\;|\;C_{1}\right)=\overset{255}{\underset{i=k+1}{\sum }}\frac{{\left(i-\mu_{1}\right)}^{2}p_{i}}{\omega_{1}}. \end{array}$$

Now, we are going to apply the discriminant analysis to evaluate and quantify the threshold at level *k*, using the measures of class separability *λ*, *κ*, and *η* based on the within-class variance *σ*_*W*_^2^, the between-class variance *σ*_*B*_^2^, and the total variance *σ*_*T*_^2^, defined as

(23) $$\begin{array}{c} \lambda =\frac{\sigma_{B}^{2}}{\sigma_{W}^{2}}\;\;\kappa =\frac{\sigma_{T}^{2}}{\sigma_{W}^{2}}\;\;\eta =\frac{\sigma_{B}^{2}}{\sigma_{T}^{2}},\\ \sigma_{W}^{2}=\omega_{0}\sigma_{0}^{2}+\omega_{1}\sigma_{1}^{2},\\ \sigma_{B}^{2}=\omega_{0}{\left(\mu_{0}-\mu_{T}\right)}^{2}+\omega_{1}{\left(\mu_{1}-\mu_{T}\right)}^{2}=\omega_{0}\omega_{1}{\left(\mu_{1}-\mu_{0}\right)}^{2},\\ \sigma_{T}^{2}=\overset{255}{\underset{i=0}{\sum }}{\left(i-\mu_{T}\right)}^{2}p_{i}. \end{array}$$

## 4. Results

### 4.1. Classification of 1D Filters

Our 1D filters are defined by the weighting function *w*(*m*) and the pyramidal construction is equivalent to convolving repeatedly the original signal with this weighting functions. Some of these Gaussian-like weighting functions are shown in Figure 5.

Note that the functions double in width with each level. The convolution acts as a low-pass filter with the band limit reduced correspondingly by one octave with each level. Because of this resemblance to the Gaussian density function we refer to the pyramid of low-pass images as the Gaussian pyramid. Just as the value of each node in the Gaussian pyramid could have been obtained directly by convolving a Gaussian-like equivalent weighting function with the original image, each value of this bandpass pyramid could be obtained by convolving a difference of two Gaussians with the original image. These functions closely resemble the Laplacian operators commonly used in image processing. For this reason the bandpass pyramid is known as a Laplacian pyramid. An important property of the Laplacian pyramid is that it is a complete image representation: the steps used to construct the pyramid may be reversed to recover the original image exactly. The top pyramidal level, *L*_*N*_, is first expanded and added to *L*_*N*−1_ to form *G*_*N*−1_. Then this array is expanded and added to *L*_*N*−2_ to recover *G*_*N*−2_, and so on.

The weighting function *w*(*m*) is determined by these constraints

(24) $$\begin{array}{c} \text{Symmetry:}\;w\left(m\right)=w\left(-m\right)\\ \text{Normalization:}\;\overset{2}{\underset{m=-2}{\sum }}w\left(m\right)=1\\ \text{Equal contribution:}\;\underset{m\;\text{odd}}{\sum }w\left(m\right)=\underset{m\;\text{even}}{\sum }w\left(m\right). \end{array}$$

Normalization, symmetry, and equal contribution are satisfied when

(25) $$\begin{array}{c} w\left(0\right)=a,\\ w\left(1\right)=w\left(-1\right)=\frac{1}{4},\\ w\left(2\right)=w\left(-2\right)=\frac{1}{4}-\frac{a}{2}. \end{array}$$

If the size is 5, we have the filter *w*

(26) $$\begin{array}{c} \left(w_{0}\left[k\right]\right)=\left[\frac{1}{4}-\frac{a}{2},\frac{1}{4},a,\frac{1}{4},\frac{1}{4}-\frac{a}{2}\right]\;k=1,\ldots ,5 \end{array}$$

which represents a uniparametric family of weighting functions with parameter *a*. Observe that if the size is bigger than 5, constraints (24) generate a multiparametric family of weighting functions.

Convolution is a basic operation of most signal analysis systems. When the convolution and decimation operators are applied repeatedly *n* times, they generate a new equivalent filter *w*_*n*_, whose length is

(27) $$\begin{array}{c} M_{0}=5,\\ M_{n}=2M_{n-1}+3\;n\geq 1, \end{array}$$

where *M*_0_ is the length of the initial filter *w*_0_.

**Figure 4:**
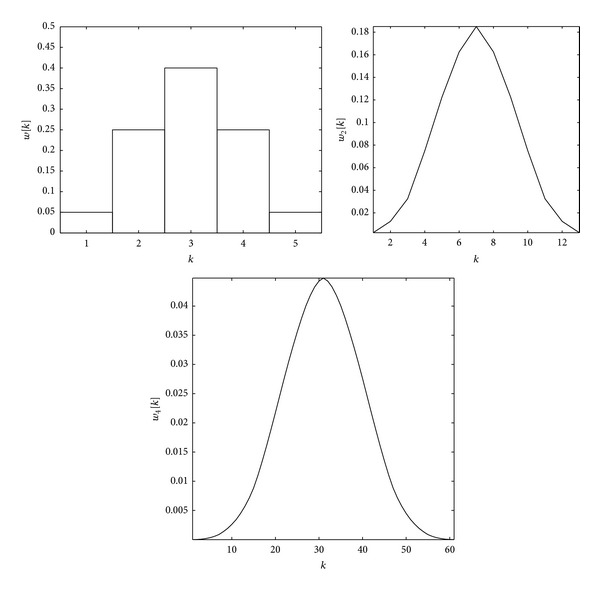
First, second, and fourth iteration of the filter 1D (*a* = 0.4).

Figure 4 shows an example of several iterations of the filter for *a* = 0.4. We can observe that there is a quick convergence to a stable shape. And, in this case, the shape of the plot of the limit filter is Gaussian.

**Figure 5:**
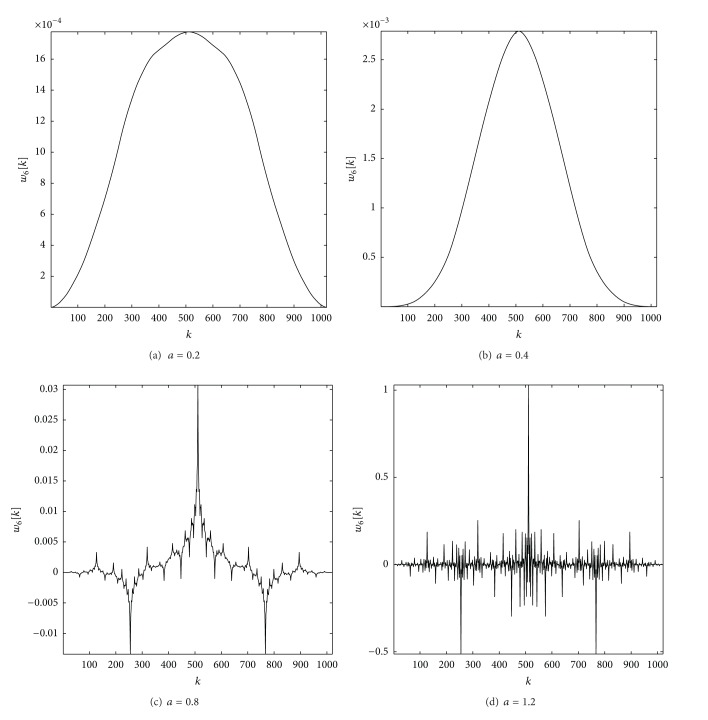
Sixth iteration of 1D Laplacian pyramid filters, *w*_6_[*k*], for different *a* values.

We have tested different values *a*, from *a* = 0.1 to *a* = 1.2. The shape of the filter, the equivalent weighting function, depends on the choice of parameter *a*. There are several different shapes for different values of *a*: Gaussian-like and fractal-like. Figure 5 shows some examples of Gaussian and fractal shapes.

The first two filters are Gaussian-like, and the last two are fractal-like. It is possible to confirm these early conclusions. We have successfully applied normality tests to verify the normality of the filters obtained with the lowest *a* values. On the other hand, in fractal geometry, the box-counting dimension is a way of determining the fractal dimension of a set. To calculate the box-counting dimension for a set *S*, we draw an evenly-spaced grid over the set and count how many boxes are required to cover the set. The box-counting dimension is calculated by applying (7).

We can see the results of fractal dimension (FD) of filters 1D whose values are shown in Table 2 and Figure 9(a). From these results and this figure we can observe a fractal behaviour when *a* < 0 and when *a* > 0.6.

The generation of bidimensional filters $\tilde{w}(m,n)$, also called generating kernels or mask filters, is based on the condition

(28) $$\begin{array}{c} \text{Separability:}\;\tilde{w}\left(m,n\right)=w\left(m\right)w\left(n\right). \end{array}$$

So, a filter $\tilde{w}(m,n)$ is called separable if it can be broken down into the convolution of two filters. This property is interesting because if we can separate a filter into two smaller filters, then usually it is computationally more efficient and quicker to apply both of them instead the original one. We work with 2D filters that can be separated into horizontal and vertical filters. 2D filters have been obtained by sequentially applying the same one-dimensional filter on rows and columns.

**Figure 6:**
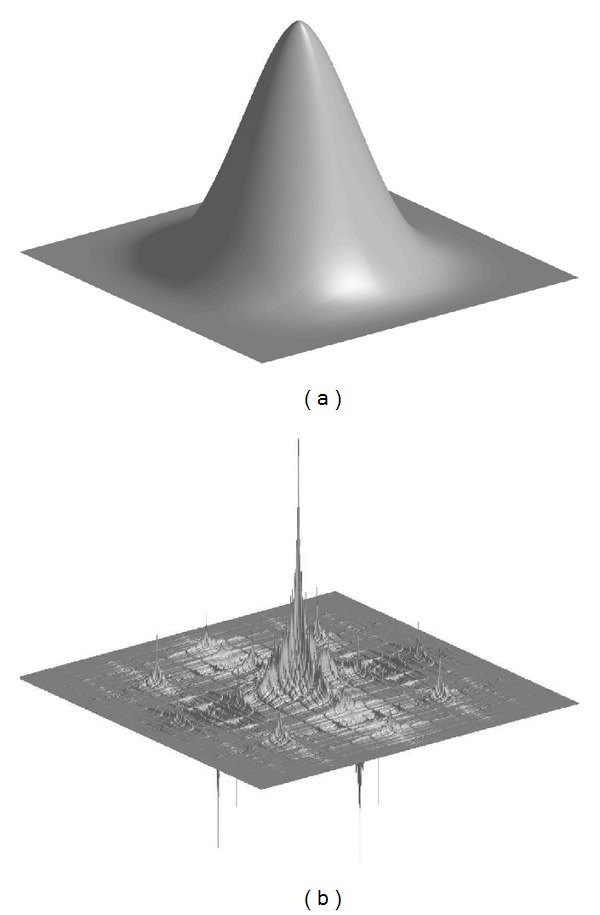
Bidimensional filters corresponding to *a* = 0.4 (a) and *a* = 0.8 (b).

When we calculate the bidimensional filters, we obtain filters like Figure 6. These are obtained when *a* = 0.4 and *a* = 0.8.

### 4.2. Normality Interval

There is a relevant result when we study the normality of filters: the Gaussian function is separable if variables are independent. Burt and Adelson show that if we choose *n* = 5 and *a* = 0.4, then the equivalent weight function is Gaussian [18]. Indeed, there is an interval for the parameter *a* where we can see the Gaussian behavior.

Once we have a chosen value for *a* and a pyramidal depth value *k*, we need to select the best normal distribution $N(\hat{\mu },\hat{\sigma })$ that fits our weights. We estimate $\hat{\mu }$ by the arithmetic mean and $\hat{\sigma }$ by the minim of all *D*_*n*_ in an empirical confidence interval calculated as

(29) CI(σ^) =(max⁡(min⁡(σ^1,σ^2)−1,0),max⁡(min⁡(σ^1,σ^2)+1,0))

with ${\hat{\sigma }}_{1}$ and ${\hat{\sigma }}_{2}$ two initial estimations of $\hat{\sigma }$.

Because the symmetry property of the filter *w*, we have that the arithmetic mean is the central point, so

(30) $$\begin{array}{c} \hat{\mu }=\frac{M_{j}+1}{2}=2^{j+1}-1 \end{array}$$

because the *j*th level is *M*_*j*_ = 2^*j*+2^ − 3, which is the solution of the linear recurrence relation (27).

Conditions to calculate ${\hat{\sigma }}_{1}$ and ${\hat{\sigma }}_{2}$ are the adjust to the histogram of the filter $\tilde{w}$ and the normal distribution related to two known percentiles; specifically,

(31) $$\begin{array}{c} \text{Pr}\left(N\left(\mu ,\sigma \right)\leq \mu -z_{1}\sigma \right)=\frac{1-p_{1}}{2},\\ \text{Pr}\left(N\left(\mu ,\sigma \right)\leq \mu -z_{2}\sigma \right)=\frac{1-p_{2}}{2} \end{array}$$

so we have the system of equations

(32) $$\begin{array}{c} {\hat{\sigma }}_{1}=\hat{\mu }-z_{1}c_{p_{1}},\\ {\hat{\sigma }}_{2}=\frac{\hat{\mu }-c_{p_{2}}}{z_{2}}, \end{array}$$

where *c*_*p*_1__ and *c*_*p*_2__ are the corresponding abscissas to percentiles *p*_1_ and *p*_2_.

**Figure 7:**
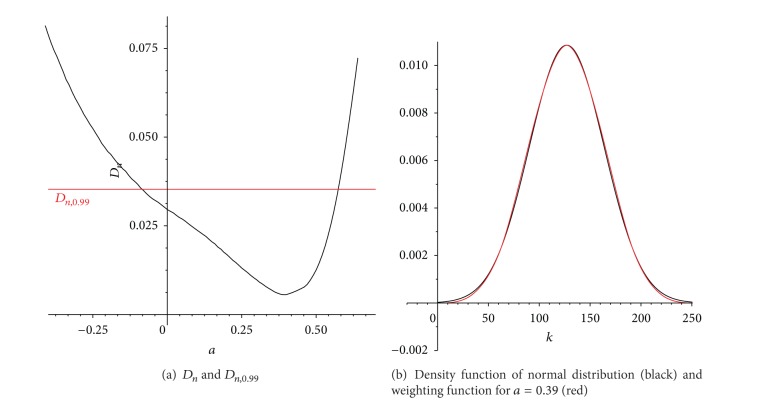
Gaussian filter adjustment.

Specifically, the numerical simulation with *n* = 5, *p*_1_ = 0.682, and *p*_2_ = 0.95 (that correspond to the normalized abscissas *z*_1_ = 1 and *z*_2_ = 1.96) generates the normality intervals, which determinates the estimation for $\hat{\sigma }$ of the normal distribution applied. Corresponding values for this case are shown in Figure 7(a). As we can see, *a* = 0.39 is the minimal value, and so it has the best adaptation to a normal distribution, as shown in Figure 7(b).

Figure 7(a) shows the value of the statistic *D*_*n*_ as a function of the parameter *a* and the normal threshold for a confidence level of 99%. For values of *a* in the interval [−0.08,0.57] the assumption of normality is not rejected. *D*_*n*_ attains the minimum for *a* = 0.39, corresponding to estimated Gaussian distribution, with mean 127 and standard deviation 36.74. Figure 7(b) shows the filter *w*_6_ and the estimated density, where we can see the adjustment goodness.

### 4.3. Image Segmentation and Performance Evaluation

**Figure 8:**
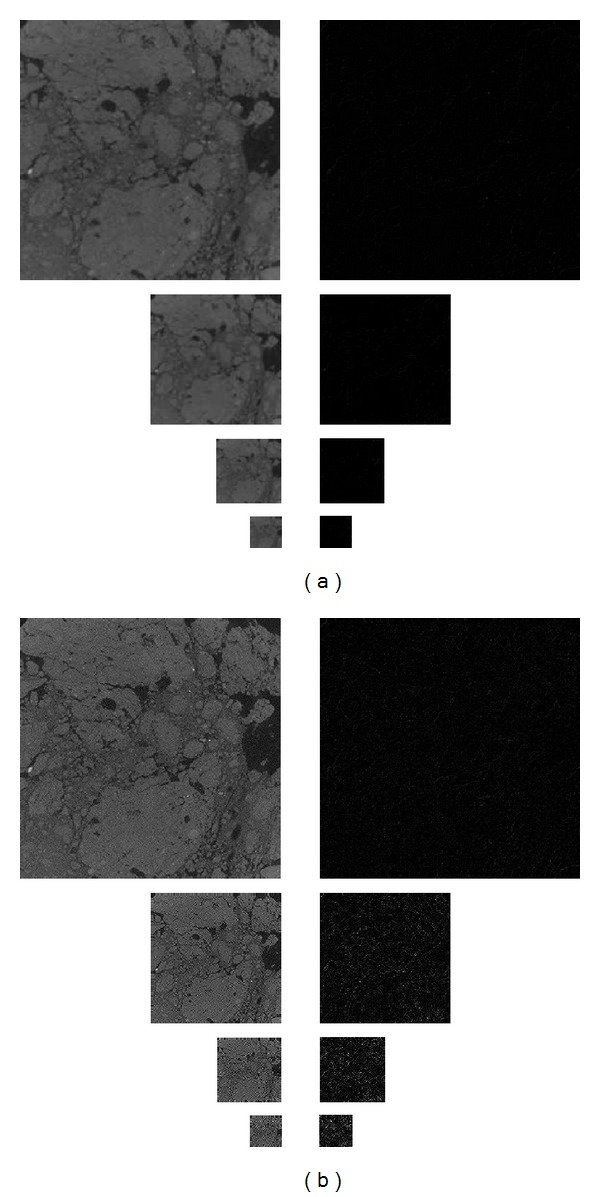
Gaussian and Laplacian pyramids of the soil image ((a) *a* = 0.4, (b) *a* = 1.2).

After these results, we have generated the Gaussian and Laplacian pyramids corresponding to one Gaussian value for *a* and another fractal value for *a*, applying the methodology previously shown, getting the corresponding Gaussian and Laplacian pyramids, and then we have compared the results obtained.  Figure 8 shows pyramidal sets for *a* = 0.4 and *a* = 1.2, corresponding to a Gaussian and a fractal, respectively.

**Figure 9:**
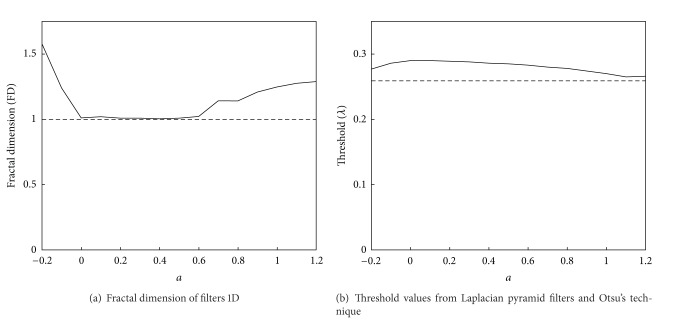
Fractal dimensions and threshold values.

**Figure 10:**
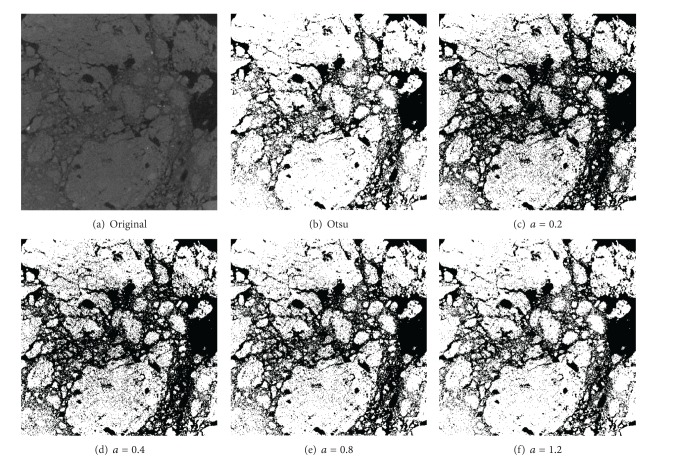
Soil image and several segmentations.

When we have applied our method to segment images with different values for the parameter *a*, we have obtained the threshold values shown in Figure 9(b) and results as shown in the Figure 10. We can compare these results with Otsu's method threshold value 0.259.

The application of our methods with different values *a* produces the results shown in Figure 10(b), together with Otsu's value. As we can see, our method obtains similar results for fractal threshold values.

### 4.4. Pore Size Distribution

**Figure 11:**
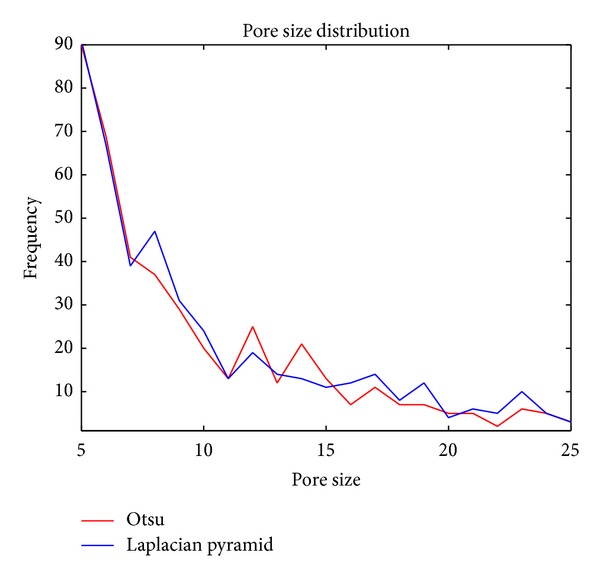
Pore size distribution.

We have presented threshold values obtained from Laplacian pyramid and the comparison with Otsu's values. On the other hand, if we compare the pore size frequency distribution obtained by Otsu's method and the threshold obtained based on Laplacian filter structure some difference is observed, as we can see in Figure 11. In the smallest size range, between 5 to 20 pixels, the former method presents higher porosity and the decrease in frequency is much smoother than with the latest method. Even the difference in porosity is not significant, Otsu's method gives 27.5% and this method estimates 31.1% the difference in sizes could affect several percolation models. These results are showed in Figure 11. Finally, the porosity obtained is this study approaches Otsu's result when *a* value increases from the range 0 till 1.2.

## 5. Conclusions

The field of fractals has been developed as an interdisciplinary area between branches of mathematics and physics and found applications in different sciences and engineering fields. In geo-information interpretation the applications developed from simple verifications of the fractal behavior of natural land structures, simulations of artificial landscapes, and classification based on the evaluation of the fractal dimension to advanced remotely sensed image analysis, scene understanding, and accurate geometric and radiometric modeling of land and land cover structures.

Referring to the computational effort, fractal analysis generally asks high complexity algorithms. Both wavelets and hierarchic representation allow now the implementation of fast algorithms or parallel ones. As a consequence a development of new experiments and operational applications is expected.

We have seen that the different choice of the parameter *a* gives two kinds of filters, the Gaussian-like and the fractal-like. This is demonstrated applying normality tests (Gaussian) and fractal dimension techniques (fractal), analytical and graphical in both cases.

The different shape of filters, Gaussian/fractal, has perceptible effects when we generate new levels of the Gaussian and Laplacian pyramids, getting blurred or accentuated new images, at every new level. Filters generated with lowest *a* values produce blurred edges and images. On the other hand, filters obtained from highest *a* values generate new images with higher contrasts and sharp edges.

Also, there is a different behaviour of the energy of the different levels of the Laplacian pyramid if we choose different *a* values, that is, if we choose a Gaussian or fractal filter. The Gaussian-like filters always make a lower energy image. This fall is slow but there is always a fall. On the other hand, fractal-like filters also fall, but this happens after several iterations, and then, the fall is bigger than the fall of the Gaussian-like filters.

These filters can be applied to image segmentation of soil images, with a simple computation and good results, quite similar or even better to some famous techniques such as Otsu's method.

Moreover, results concerning porosity are similar but there are differences in pore size distribution that could improve percolation simulations. The implementation of this method in three dimensions is straightforward.

Future work could add other image segmentation techniques and neural networks methods to select the optimal threshold values from information and characteristics of the image.

## Figures and Tables

**Table 1:** Range of porosity values.

Unconsolidated deposits	Porosity	Rocks	Porosity
Gravel	0.25–0.40	Fractured basalt	0.05–0.50
Sand	0.25–0.50	Karst limestone	0.05–0.50
Silt	0.35–0.50	Sandstone	0.05–0.30
Clay	0.40–0.70	Limestone, dolomite	0.00–0.20
		Shale	0.00–0.10
		Fractured crystalline rock	0.00–0.10
		Dense crystalline rock	0.00–0.05

**Table 2:** Fractal dimension of filters.

*a*	Fractal dimension
−0.2	1.578
−0.1	1.240
0.0	1.011
0.1	1.020
0.2	1.009
0.3	1.009
0.4	1.004
0.5	1.009
0.6	1.022
0.7	1.142
0.8	1.141
0.9	1.209
1.0	1.248
1.1	1.276
1.2	1.289
